# Developmental, but not Homeostatic, Collagen V Expression Regulates Mature Murine Supraspinatus Tendon Structure, Function, and Gene Expression

**DOI:** 10.1002/jor.70164

**Published:** 2026-02-23

**Authors:** Michael S. DiStefano, Jeremy D. Eekhoff, Stephanie N. Weiss, Courtney A. Nuss, Rebecca L. Betts, Andrew F. Kuntz, Louis J. Soslowsky

**Affiliations:** ^1^ McKay Orthopedic Laboratory University of Pennsylvania Philadelphia Pennsylvania USA; ^2^ Department of Bioengineering University of Pennsylvania Philadelphia Pennsylvania USA

**Keywords:** collagen V, structure‐function, supraspinatus tendon

## Abstract

The development of tendon hierarchical structure is dependent on collagen I assembly into fibrils and higher‐order assemblies, a process regulated by interactions involving collagen V, a quantitatively minor yet essential component of the tendon extracellular matrix. Collagen V critically regulates fibrillogenesis and is expressed throughout tendon development and maturation. Clinically, deficiency of collagen V manifests as classic Ehlers‐Danlos syndrome (cEDS), a disorder characterized by hyperextensible skin, joint hypermobility and instability, and impaired wound healing. Recent studies in mouse supraspinatus tendons—which experience complex, region‐dependent loading environments at insertion and midsubstance—demonstrate that developmental collagen V deficiency results in altered tendon structure and biomechanical function. However, differences in structure, mechanics, and gene expression resulting from reduced collagen V expression from embryonic development versus reduction during homeostasis have not been clearly delineated in mature tendons. To address this gap, this study elucidated the role of collagen V on region‐specific tendon structural, functional, and compositional properties in mature, day 150 supraspinatus tendons. Tendon‐targeted *Col5a1* deficiency from embryonic development resulted in substantial structural, mechanical, and transcriptional alterations, including disrupted fibril organization, compromised mechanical properties, and altered gene expression profiles. Conversely, acute deficiency and knockdown of *Col5a1* in mature tendons resulted in relatively minimal changes. Collectively, these findings identify a distinct and critical role for collagen V during tendon development, rather than homeostasis, in establishing region‐specific multiscale structural, mechanical, and molecular properties essential for mature supraspinatus tendon function.

## 1 Introduction

Tendons are specialized connective tissues that transmit force from muscle to bone and contribute to joint stability. Their ability to withstand high loads depends on a complex hierarchical structure composed primarily of collagen type I [1]. Collagen molecules assemble into fibrils, which bundle into fibers, fascicles, and ultimately the full tendon. This structure enables tendons to respond dynamically to load through collagen fibril uncrimping, realignment, sliding, and deformation [2]. Although collagen I is the predominant structural component, several quantitatively minor matrix constituents—including proteoglycans and minor collagens—are critical for establishing and maintaining tendon organization and function [3, 4, 5, 6, 7].

Among these minor collagens, collagen V plays an essential regulatory role despite representing only ~2% of the total fibril‐forming collagen content in mature tendon [8]. Collagen V is a fibrillar collagen and is incorporated into the core of heterotypic fibrils with collagen I and is required for proper fibril nucleation and lateral fibril growth to maintain homeostasis during development and maturation [9]. Clinically, mutations in COL5A1 are associated with classic Ehlers–Danlos syndrome (EDS), a heritable connective tissue disorder characterized by skin hyperextensibility, joint hypermobility, and poor wound healing [10]. Previous studies demonstrated the role of collagen V on structural and mechanical properties of murine supraspinatus (a largely intrasynovial) and patellar (a largely extrasynovial) tendons [5, 11, 12, 13]. Moreover, the role of collagen V on structural and functional properties of these tissues is not limited to development—collagen V is upregulated following injury in tendons and ligaments, indicating a role in matrix remodeling and repair. We previously demonstrated that collagen V haploinsufficiency (*Col5a1* ^+/^^−^ mouse model) results in delayed healing in our murine patellar tendon injury model compared to wild‐type control tendons [13]. Furthermore, we showed that inducible knockdown of Collagen V at the time of injury in our murine patellar tendon injury model results in dysregulated expression of several genes throughout the healing response and altered collagen fibril diameter [14]. However, the differential roles of collagen V on the development of tendon structure, function, and gene expression versus its role in regulating these properties after establishment of the tendon matrix remain unknown. Therefore, the objectives of this study were to (1) elucidate the role of collagen V in supraspinatus tendon development, and (2) determine the homeostatic role of collagen V on supraspinatus tendon structure and function.

We hypothesized that: (1) due to the role of collagen V in regulating tendon structure during development, collagen V heterozygous and knockout supraspinatus tendons would exhibit inferior whole‐tissue and regional elastic mechanical properties, as well as altered regional collagen fiber realignment, fibril diameter distributions, and gene expression profiles compared to wild‐type control tendons; and (2) because tendon hierarchical structure is well‐established by maturity, acute knockdown of collagen V in adult tendons would result in minimal changes to regional mechanical properties, collagen fiber realignment, fibril diameter distributions, and gene expression relative to wild‐type control tendons.

This study provides new insights into the distinct temporal regulatory roles of collagen V in tendon development and homeostasis. By combining developmental and inducible knockout models, we aim to elucidate the roles of collagen V in early matrix assembly versus its function in the maintenance and adaptation of mature tendon.

## 2 Methods

### 2.1 Animal Use and Study Design

This study was approved by the University of Pennsylvania Institutional Animal Care and Use Committee (IACUC; protocol 806203) and carried out in strict accordance with guidelines established in the *Guide for the Care and Use of Laboratory Animals*. C57BL/6 male mice were housed in an AAALAC accredited facility that maintained 12‐h light/dark cycles, temperatures between 20°C and 26°C and humidity between 30% and 70%. Five different mouse genotypes were used in this study. First, tendon‐targeted scleraxis (Scx)‐Cre;*Col5a1* ^flox/flox^ (TEN‐KO), Scx‐Cre;*Col5a1* ^flox/wt^ (TEN‐HET), and Cre‐ littermate control (WT) mice were used as described [5, 8, 11, 12]. Second, tamoxifen (TM)‐inducible ROSA26‐CreER^T2^; *Col5a1* ^flox/+^ and ROSA26‐CreER^T2^;*Col5a1* ^flox/flox^ mice were used, as described [14]. At 120 days old, inducible mice received 3 consecutive daily TM injections (100 mg/kg) to induce Cre‐mediated excision of floxed *Col5a1* alleles, resulting in inducible *Col5a1* + /− (I‐HET) and *Col5a1*‐/‐ (I‐NULL) genotypes which were compared to wild‐type (WT) controls. All mice were sacrificed at postnatal (P) day 150 (~5 months). Supraspinatus tendons were evaluated for whole‐tissue and site‐specific (insertion vs. midsubstance) mechanics and collagen fiber realignment (*n* = 10/group), site‐specific atomic force microscopy (*n* = 8–11/group), fibril morphology (*n* = 5/group), site‐specific cellularity and cell shape (n = 6/group), and site‐specific gene expression (*n* = 4/group). Both limbs from each mouse were randomly allocated to different assays, ensuring that no two limbs from the same animal were assigned to the same assay. All assays were conducted by blinded investigators.

### 2.2 Biomechanical Testing and Collagen Fiber Realignment Analysis

Mice designated for biomechanical testing were frozen at − 20°C until the day of testing. Mice were thawed at room temperature and the supraspinatus tendon‐humerus complex from each mouse was carefully dissected to remove extraneous tissue. Stain lines were applied for optical tracking at 0 mm, 1 mm, 2 mm, and 2.5 mm from the humeral insertion, where the insertion region was defined as the area between 0 and 1 mm from the humeral insertion and the midsubstance region was between 1 and 2 mm from the humeral insertion [5, 11, 12, 15, 16]. A custom laser device [17] was used to measure cross‐sectional area of the supraspinatus tendon. The free end of the tendon was placed between two sandpaper tabs adhered with cyanoacrylate glue to prevent slippage. The humerus was secured in a custom construct with polymethyl methacrylate, and the construct was mounted on a material testing machine (Instron 5848; Instron, Norwood, MA) with a 10 N load cell. All testing was conducted in a phosphate buffered saline bath at 37°C. Each sample was first preloaded to 0.025 N [16]. The testing protocol began with 10 cycles of preconditioning between 0.5% and 1.5% grip strain at 0.25 Hz. This was followed by three stress relaxation tests, consisting of ramps to 3%, 5%, and 7% grip strain at a rate of 5% strain per second, with 10‐min holds after each ramp. After each stress relaxation, frequency sweeps were conducted, involving 10 cycles of 0.125% sinusoidal grip strain at 0.1, 1, 5, and 10 Hz. Following a 10‐min rest at zero displacement, a quasistatic ramp‐to‐failure at a strain rate of 0.1% s^−1^ was completed [5, 12, 15, 16]. Data were collected at 100 Hz. Elastic parameters maximum load and stiffness and maximum stress were computed from the force‐displacement and stress‐strain data, respectively. Viscoelastic parameters stress relaxation, dynamic modulus, and the phase angle were quantified for each stress relaxation and frequency sweep. Images were captured during the quasistatic ramp‐to‐failure and used to optically track the insertion and midsubstance regions. Using a custom script (MATLAB; Natick, MA), optical Lagrangian strain was quantified for each region and was used to calculate insertion and midsubstance moduli, as described [16]. Collagen fiber realignment was also quantified from the ramp‐to‐failure images using cross‐polarization imaging. Quantified regional fiber realignment data, represented as circular variance, were normalized to the first discrete data point at 0% strain and interpolated in MATLAB using a polynomial fit as a function of strain from the load‐displacement data [15, 16, 18].

### 2.3 Atomic Force Microscopy

Supraspinatus tendon‐humerus complexes were prepared for mechanical testing as described above. Samples were subjected to 10 cycles of preconditioning from 0% to 1% strain at 1 Hz followed by a 1‐min rest and then a ramp to a randomly assigned strain (0%, 3%, or 6%) at a strain rate of 0.1% s^−1^. These applied strain values were chosen based on the initial, toe, and linear regions of the load‐displacement curves from tendons mechanically tested in this study. Supraspinatus tendons were immediately flash frozen using rapid freeze spray (Azer Scientific, Morgantown, PA) after reaching the target strain, removed from the test fixture and embedded in optimal cutting temperature compound while keeping the tissue frozen to maintain the applied strain [2, 16]. Cryosections of the tendons were collected at 20 μm thickness, fixed in 10% neutral buffered formalin, and air dried. Collagen fibrils within the strained sections were imaged using an atomic force microscope (BioScope Catalyst; Bruker, Billerica, MA) equipped with a VTESPA‐300 cantilever tip (Bruker) using tapping mode in air. Five 2 × 2 μm images at 512 × 512 pixel resolution were acquired in both the insertion region and midsubstance region across multiple tissue sections for each sample. After rough extrafibrillar topography was removed using polynomial background subtraction, collagen fibril d‐period was measured using Fourier transform analysis with a custom MATLAB script. The average d‐period length, local variance (average variance in d‐period length within individual images) and global variance (variance in d‐period length across entire sample) were calculated for the insertion and midsubstance regions of each sample [16].

### 2.4 Cell Density and Nuclear Aspect Ratio

For quantification of cellularity and nuclear aspect ratio, whole shoulders were grossly harvested and immediately fixed, decalcified, and processed for paraffin embedding using standard histological techniques [15, 16]. Briefly, tendons were sectioned at 7 µm thickness in the coronal plane, stained with DRAQ5 (Thermo, Waltham, MA) and imaged at 20x magnification. One representative region of interest was selected from the insertion and one from the midsubstance for nuclear segmentation using Otsu's thresholding method to quantify cellularity and nuclear aspect ratio (CellProfiler 4) [19].

### 2.5 Collagen Fibril Morphology

Tendons assigned for fibril morphology analysis using transmission electron microscopy (TEM) were prepared as described [20]. Following euthanasia, supraspinatus tendons were fixed in Karnovsky's fixative (4% paraformaldehyde, 2.5% glutaraldehyde, 0.1 M sodium cacodylate, 8.0 mM calcium chloride), post‐fixed and stained with 1% osmium tetroxide, dehydrated in ethanol and embedded in Epon resin. Prior to embedding, tendons were separated into insertion and midsubstance regions for analysis. Tendons were then sectioned in the transverse plane and ultrathin sections (60–80 nm) were stained with UranyLess (EMS 22409) and 1% phosphotungstic acid. Images were acquired at 60,000x magnification using a JEOL 1010 transmission electron microscope. For each sample, a set of 15 images were acquired and fibril diameters from ten randomly selected digital images from this set were measured using a custom MATLAB script, as previously described [16].

### 2.6 Gene Expression Analysis

Following euthanasia, supraspinatus tendons were dissected, flash frozen in liquid nitrogen, and stored at − 80°C. Tendons were later thawed in RNAlater‐ICE (Invitrogen, Carlsbad, CA), carefully separated into their insertion and midsubstance regions using a custom fixture [15, 16], transferred to a phenol‐based lysis reagent, TRIzol (Invitrogen), and mechanically homogenized using a pestle. RNA was then isolated (Direct‐zol RNA Microprep; Zymo, Irvine, CA), converted to cDNA (High‐Capacity cDNA RT, Thermo), and preamplified for 15 cycles using 96 pre‐selected probes (TaqMan, Thermo) for gene expression analysis (Supporting Information Table S1). Preamplified cDNA was loaded into a Standard BioTools 96.96 Dynamic Array IFC (BMK‐M‐96.96, Standard BioTools, South San Francisco, CA) and run with the 96 selected gene panel primers. Target genes included those of collagens, non‐collagenous matrix, inflammatory/remodeling, cell‐cell/cell‐matrix, cell signaling, and cell markers/cell cycle. Cycle threshold (Ct) values were evaluated for each gene, including for the housekeeper genes *Rn18s*, *Abl1*, and *Rps17*. ΔCt values were computed by subtracting gene Ct values from the average housekeeper Ct value for that sample.

### 2.7 Statistics

Data points considered statistical outliers, identified as values falling below 1.5 times the interquartile range from the first quartile or above 1.5 times the interquartile range from the third quartile, were excluded from analysis (Supporting Information Table S2). Data distributions were tested for normality using Shapiro–Wilk tests. Comparisons between whole‐tissue mechanical properties, regional moduli, regional collagen fiber realignment at each strain level, and regional cell density and cell shape were made using one‐way ANOVAs with Tukey‐adjusted post‐hoc comparisons between the WT, TEN‐HET and TEN‐KO groups as well as between the WT, I‐HET, and I‐NULL groups where appropriate. For collagen fibril deformation, and local and global variation, genotype data for the insertion and midsubstance regions were compared using Student's t‐tests. Collagen fibril diameter distributions were compared within the insertion and midsubstance regions using Kolmogorov‐Smirnov tests. Volcano plots were generated to compare expression of target genes between genotypes, with statistical significance determined using Student's t‐tests. Genes with both significant *p*‐values (*p* < 0.05) and log₂ fold changes > 1 or < −1 were identified as differentially upregulated or downregulated, respectively. Statistical comparisons were not made between the conditional and inducible models due to the use of different Cre drivers (Scx‐Cre vs. ROSA26‐CreER^T2^).

## 3 Results

### 3.1 *Col5a1* Expression

**Figure 1:**
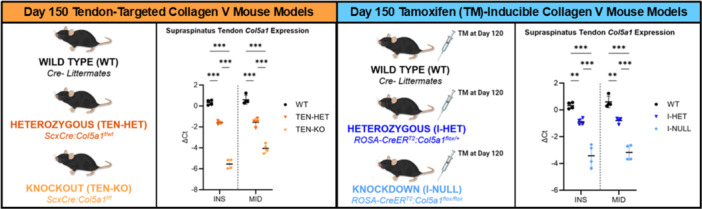
Day 150 tendon‐targeted (TEN‐HET and TEN‐KO) and tamoxifen(TM)‐inducible (I‐HET and I‐NULL) collagen V mouse models demonstrated dose‐dependent decreases in *Col5a1* expression in both the insertion (INS) and midsubstance (MID) regions of supraspinatus tendons relative to WT controls. Data are presented as mean ± standard deviation (*p ≤ 0.05, **p ≤ 0.01, ***p ≤ 0.001).

In both the insertion (INS) and midsubstance (MID) regions, substantial dose‐dependent knockdown of *Col5a1* expression was observed in the tendon‐targeted (Scx‐Cre) TEN‐HET and TEN‐KO tendons relative to WT tendons and in the TM‐inducible (ROSA26‐CreER^T2^) I‐HET and I‐NULL supraspinatus tendons relative to WT tendons (Figure 1).

### 3.2 Whole‐Tendon Mechanics

**Figure 2:**
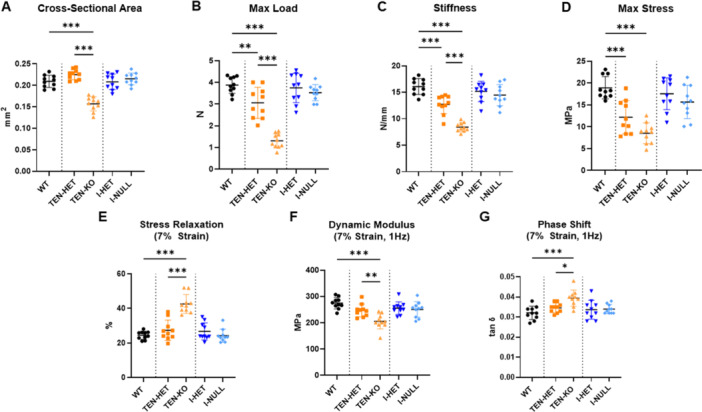
TEN‐KO tendons exhibited smaller (A) cross‐sectional area relative to WT and TEN‐HET tendons. TEN‐HET and TEN‐KO tendons demonstrated reduced (B) max load, (C) stiffness, and (D) max stress relative to WT tendons. TEN‐KO tendons show increased (E) stress relaxation, reduced (F) dynamic modulus, and increased (G) phase shift relative to WT and TEN‐HET tendons. (7% strain, 1 Hz data shown). I‐HET and I‐NULL tendons did not demonstrate any differences in these elastic and viscoelastic properties relative to WT tendons. Data are presented as mean ± standard deviation (*p ≤ 0.05, **p ≤ 0.01, ***p ≤ 0.001).

Whole‐tendon cross‐sectional area was reduced in the TEN‐KO group compared to the TEN‐HET and WT groups (Figure 2A). Collagen V deficiency and knockout resulted in dose‐dependent reductions in max load, linear stiffness, and max stress (Figure 2B–D). Viscoelastic differences were also observed. Percent relaxation was increased in TEN‐KO tendons compared with TEN‐HET and WT tendons at all strain levels (7% strain shown in Figure 2E). Additionally, collagen V TEN‐HET and TEN‐KO resulted in dose‐dependent reductions in dynamic modulus, while phase shift was increased in TEN‐KO tendons relative to TEN‐HET and WT across all strain levels and frequencies (7% strain at 1 Hz shown in Figure 2F,G). No differences were observed in elastic mechanical properties, max load and linear stiffness, and viscoelastic properties, percent relaxation, dynamic modulus, and phase shift, with acute collagen V knockdown (I‐HET and I‐NULL).

### 3.3 Site‐Specific Mechanics

**Figure 3:**
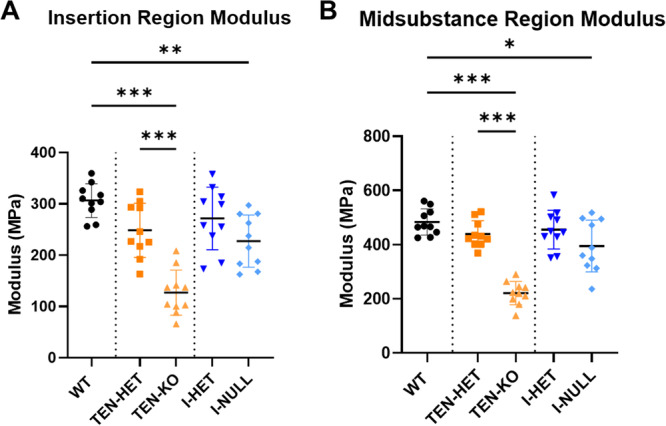
TEN‐KO tendons demonstrated reduced (A) insertion and (B) midsubstance modulus relative to WT and TEN‐HET tendons. I‐NULL tendons exhibited reduced (A) insertion and (B) midsubstance modulus relative to WT tendons. Data are presented as mean ± standard deviation (*p ≤ 0.05, **p < 0.01, ***p ≤ 0.001).

In the insertion region, TEN‐KO tendons demonstrated reduced modulus relative to TEN‐HET and WT controls. Additionally, I‐NULL tendons exhibited reduced modulus relative to WT tendons in the insertion region. (Figure 3A). In the midsubstance region, TEN‐KO exhibited decreased modulus compared to TEN‐HET and WT tendons, and I‐NULL tendons demonstrated reduced modulus relative to WT tendons (Figure 3B).

### 3.4 Site‐Specific Collagen Fiber Realignment

**Figure 4:**
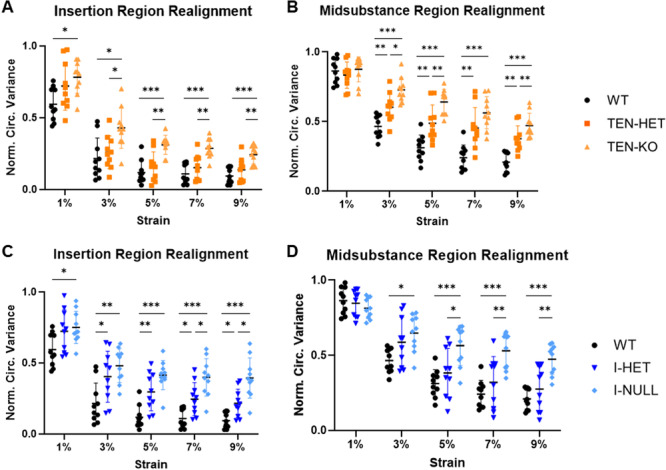
TEN‐KO tendons demonstrated increased normalized circular variance, indicative of decreased collagen reorganization, relative to TEN‐HET and WT tendons in the (A) insertion region across all strain levels. TEN‐HET and TEN‐KO tendons exhibited increased normalized circular variance relative to WT tendons from 3% to 9% strain in the (B) midsubstance region. I‐HET and I‐NULL tendons demonstrated increased normalized circular variance relative to WT tendons, notably from 3% to 9% strain in the (C) insertion region whereas I‐NULL tendons demonstrated increased normalized circular variance relative to I‐HET and WT tendons in the (D) midsubstance region. Data are presented as mean ± standard deviation (*p ≤ 0.05, **p ≤ 0.01, ***p ≤ 0.001).

TEN‐HET and TEN‐KO tendons demonstrated decreased collagen fiber realignment relative to WT controls, as demonstrated by greater normalized circular variance values in the insertion and midsubstance regions (Figure 4A–B). Furthermore, I‐HET and I‐NULL tendons exhibited reduced collagen fiber realignment relative to WT tendons in the insertion region from 3% to 9% strain (Figure 4C). In the midsubstance region, I‐NULL tendons demonstrated decreased collagen fiber realignment relative to I‐HET and WT‐tendons (Figure 4D).

### 3.5 Site‐Specific Atomic Force Microscopy

**Figure 5:**
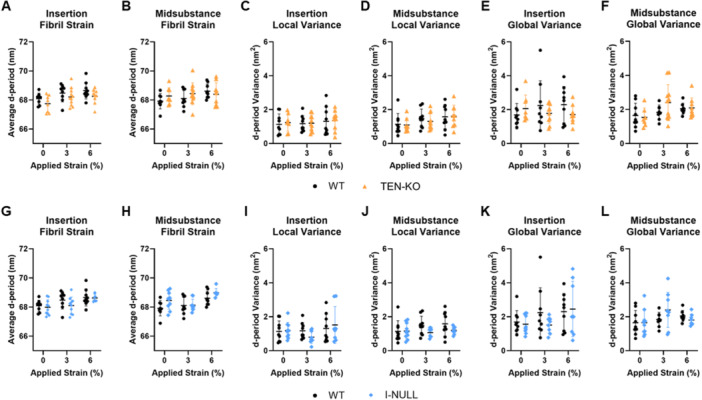
No differences were observed between TEN‐KO and WT in (A) insertion and (B) midsubstance fibril strain, (C) insertion and (D) midsubstance local variance, and (E) insertion and (F) midsubstance global variance at 0%, 3%, and 6% applied strain. Similarly, I‐NULL tendons did not demonstrate differences relative to WT tendons in (G) insertion and (H) midsubstance fibril strain, (I) insertion and (J) midsubstance local variance, and (K) insertion and (L) global variance at 0%, 3%, and 6% applied strain. Data are presented as mean ± standard deviation.

TEN‐KO tendons did not demonstrate any differences relative to WT tendons across all applied strain levels for insertion and midsubstance fibril strain (Figure 5A–B), local variance (Figure 5C–D), and global variance (Figure 5E–F). Similarly, I‐NULL tendons did not exhibit any differences relative to WT tendons across all applied strain levels for insertion and midsubstance fibril strain (Figure 5G–H), local variance (Figure 5I–J), and global variance (Figure 5K–L).

### 3.6 Site‐Specific Cell Density and Nuclear Aspect Ratio

**Figure 6:**
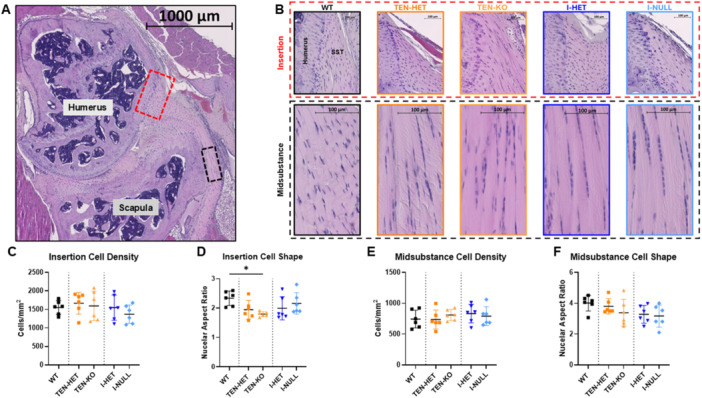
(A) Representative H&E coronal image of the supraspinatus tendon within the rotator cuff with the insertion (red dotted box) and midsubstance (black dotted box) regions demarcated. (B) Representative H&E images of the insertion and midsubstance regions of the supraspinatus tendons (SST) across genotypes. In the insertion region, no differences were observed in (C) cell density across genotype. However, TEN‐KO tendons demonstrated decreased (D) nuclear aspect ratio, indicative of more rounded cells, relative to WT tendons. In the midsubstance region, no differences were observed in (E) cell density or (F) nuclear aspect ratio across genotype. Data are presented as mean ± standard deviation (*p ≤ 0.05).

H&E staining did not reveal morphological differences in the supraspinatus tendon across genotypes (Figure 6A). Further, in both the insertion and midsubstance regions (Figure 6B), no differences were observed across genotype in cell density (Figure 6C,E). However, TEN‐KO tendons demonstrated decreased nuclear aspect ratio, indicative of more rounded cells, in the insertion region relative to WT tendons (Figure 6D). No differences were observed in nuclear aspect ratio across genotype in the midsubstance region (Figure 6F).

### 3.7 Site‐Specific Fibril Morphology

**Figure 7:**
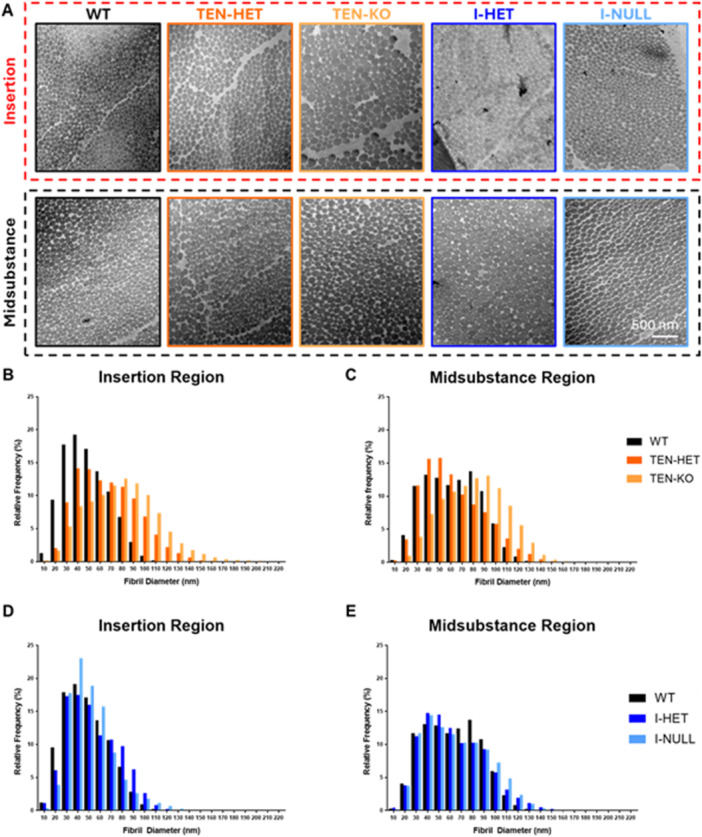
Representative TEM images (A) demonstrate shifts towards larger diameter fibrils in TEN‐HET and TEN‐KO tendons relative to WT tendons in the (B) insertion region (*p* < 0.001). In the (C) midsubstance region, TEN‐KO tendons exhibited a shift toward larger diameter fibrils compared to WT and TEN‐HET tendons (*p* < 0.001). I‐HET and I‐NULL tendons demonstrated subtle shifts towards larger diameter fibrils relative to WT tendons in the (D) insertion and (E) midsubstance regions (*p* < 0.001).

Representative TEM images (Figure 7A) demonstrate shifts towards larger diameter fibrils in TEN‐HET and TEN‐KO tendons relative to WT tendons (*p* < 0.001) in the insertion region (Figure 7B). In the midsubstance region, TEN‐KO tendons exhibited a shift toward larger diameter fibrils compared to WT and TEN‐HET tendons (*p* < 0.001) (Figure 7C). Less noticeable changes were observed in the tamoxifen‐inducible tendons. I‐HET and I‐NULL tendons demonstrated a shift towards larger diameter fibrils relative to WT tendons in the insertion (Figure 7D) and midsubstance (Figure 7E) regions (*p* < 0.001).

### 3.8 Site‐Specific Gene Expression

**Figure 8:**
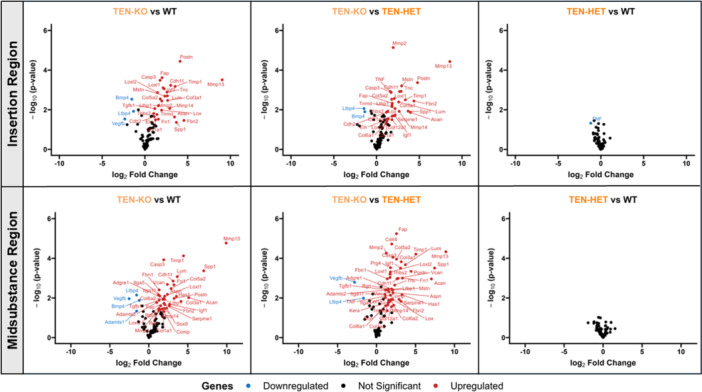
Volcano plots display differential gene expression in the insertion (top) and midsubstance (bottom) regions of supraspinatus tendons. TEN‐KO tendons show the greatest number of changes relative to WT and TEN‐HET tendons, in both the insertion and midsubstance regions, while TEN‐HET tendons show minimal differences compared to WT tendons.

**Figure 9:**
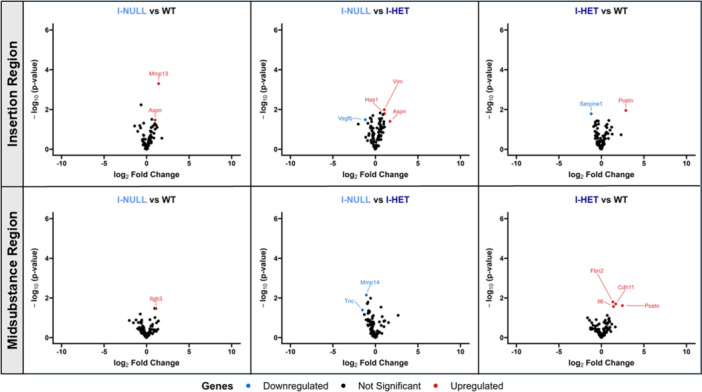
Volcano plots show differential gene expression in the insertion (top) and midsubstance (bottom) regions of supraspinatus tendons. Minimal changes in gene expression were observed across WT, I‐HET, and I‐NULL tendons.

Gene expression analysis revealed robust changes in TEN‐KO tendons relative to TEN‐HET and WT tendons in the insertion and midsubstance regions (Figure 8). In the insertion and midsubstance regions, TEN‐KO tendons showed upregulation of extracellular matrix remodeling enzymes including *Mmp2*, *Mmp13*, *Mmp14*, *Timp1*, matricellular proteins, *Postn*, *Fap*, *Fn1*, *Spp1*, *Tnc*, *Lum*, and critical tendon‐related collagens such as *Col1a1* and *Col3a1*. Other elevated genes included *Lox*, *Loxl1*, *Loxl2*, *Casp3*, *Igf1*, and *Cdh11*. Downregulated genes in TEN‐KO tendons included *Bmp4*, *Ltbp4*, and *Vegfb*. Minimal differences in gene expression were observed between WT and TEN‐HET tendons, with TNF‐α downregulated in the insertion region of TEN‐HET tendons relative to WT tendons. Minor gene expression changes were observed between WT, I‐HET, and I‐NULL tendons in the insertion and midsubstance regions. *Aspn* was upregulated in the insertion region of I‐NULL tendons relative to WT and I‐HET tendons and *Postn* was upregulated in I‐HET tendons relative to WT tendons in both the insertion and midsubstance regions (Figure 9).

## 4 Discussion

In accordance with our hypothesis, tendon‐targeted knockout of collagen V from development resulted in decreased cross‐sectional area, failure load, and stiffness, alongside altered viscoelastic properties such as increased stress relaxation and phase shift and decreased dynamic modulus. When assessing regional mechanics, we observed that both the insertion and midsubstance regions of TEN‐KO tendons exhibited reduced modulus compared to WT and TEN‐HET tendons. These results underscore the critical role of collagen V in establishing the mechanical integrity of the supraspinatus tendon, as supported by previous studies [5, 8, 12]. The observed reduction in cross‐sectional area suggests reduced tissue growth or ECM deposition, which may result from disrupted fibrillogenesis during development [8, 21, 22]. In contrast, acute knockdown of collagen V in adult mice (I‐NULL and I‐HET) did not produce changes in whole‐tendon elastic and viscoelastic mechanical properties. I‐NULL tendons demonstrated reduced insertion and midsubstance modulus relative to WT controls, suggesting that collagen V may contribute to maintaining the mechanical integrity of the supraspinatus tendon during maturation.

Collagen fiber realignment was also altered by collagen V deficiency and knockout. TEN‐KO and TEN‐HET tendons exhibited decreased fiber realignment across all strain levels in both the insertion and midsubstance, as evidenced by increased circular variance. This suggests that developmental collagen V loss leads to persistent deficits in realignment of collagen fibers under mechanical load, possibly due to abnormal fibril nucleation and initial assembly [5, 8, 11, 12]. Notably, the reduction in collagen fiber realignment in response to load in the TEN‐HET and TEN‐KO tendons may contribute to the reductions observed in elastic and viscoelastic properties observed in these same tendons. In the inducible model, I‐NULL tendons showed reduced collagen fiber realignment compared with WT tendons, with more notable differences present in the insertion region, which may reflect a diminished ability of the adult tendon to undergo microstructural reorganization when collagen V is acutely lost. The presence of any realignment deficits in the inducible model suggests that collagen V may contribute to maintaining fiber reorganization behavior under load and may explain the reductions observed in both the insertion and midsubstance region moduli of I‐NULL tendons.

Despite these large‐scale mechanical and structural deficits, atomic force microscopy revealed that fibril‐level deformation under load was largely preserved across genotypes. Measurements of collagen d‐period strain, local variance, and global variance showed no significant differences between WT, TEN‐KO, and I‐NULL tendons in either region or at any applied strain level. This preservation of nanoscale mechanical behavior implies that once collagen fibrils are formed, their structural behavior under applied loading is not affected by the absence of collagen V. These findings support the idea that collagen V is a critical regulator of collagen fibrillogenesis but may not be required for the ongoing structural and functional stability of individual fibrils. However, it is important to note that while individual fibrils remained functionally intact, the larger architectural organization and fiber network behavior were clearly disrupted, as evidenced by the reduced mechanical properties and collagen fiber realignment data in the TEN‐KO tendons. This underscores the hierarchical nature of tendon structure‐function relationships, where nanoscale structural integrity alone is insufficient to ensure tissue‐level mechanical performance.

Transmission electron microscopy confirmed that collagen V is essential for regulating fibril morphology during development. In the insertion region, TEN‐HET and TEN‐KO tendons demonstrated shifts towards larger diameter fibrils relative to WT tendons. In the midsubstance, only TEN‐KO tendons demonstrated this shift towards larger diameter fibrils relative to WT and TEN‐HET tendons. These findings align with prior in vitro studies demonstrating that collagen V acts as a nucleator that limits fibril diameter by capping growing fibrils during early assembly [23, 24]. Inducible knockdown produced only subtle shifts in fibril diameter in I‐HET and I‐NULL tendons, reinforcing the idea that fibril diameter is primarily established during early matrix development and remains relatively stable thereafter. Nonetheless, the TEM data provide strong evidence that collagen V's role in fibrillogenesis is tightly linked to the developmental window which may have contributed to the reductions in mechanical properties and alterations in realignment observed in this study.

Histological analysis revealed largely preserved cellularity and nuclear morphology across genotypes, indicating that loss of collagen V, whether developmental or inducible, does not substantially alter tenocyte density or gross morphology in mature tendons. Quantification of cell density showed no differences across genotypes in either the insertion or midsubstance regions, suggesting that collagen V may not be required for maintaining tenocyte numbers during development or adulthood. This aligns with prior studies showing that cell density is relatively stable in adult tendon under homeostatic conditions. However, assessment of nuclear aspect ratio, a proxy for cell shape, revealed more rounded nuclei in the insertion region of TEN‐KO tendons compared to WT. This finding may be indicative of impaired matrix organization and reduced mechanical loading at the cell‐matrix interface. Rounded nuclei are often associated with lower cytoskeletal tension and softer or more disorganized matrices, suggesting that collagen V deficiency during development may disrupt matrix cues that guide tenocyte shape and alignment [25, 26, 27]. These changes were not observed in the inducible I‐HET and I‐NULL tendons, reinforcing the idea that nuclear morphology may be dependent more on changes in matrix architecture during tendon development, rather than in response to acute reduction of collagen V at maturation. Overall, the histological findings support the concept that collagen V regulates tendon structure through its effects on extracellular matrix organization rather than through direct modulation of cell density or morphology.

TEN‐KO tendons demonstrated extensive changes in gene expression relative to WT and TEN‐HET tendons. These gene expression changes reveal coordinated alterations in pathways involved in matrix degradation, fibril assembly, crosslinking, mechanosensing, and growth factor signaling, suggesting that collagen V deficiency disrupts not only structural matrix integrity but also the signaling environment required for tendon homeostasis. Among the most strongly upregulated genes in the TEN‐KO insertion was *Mmp13*. *Mmp13* is a well‐established mediator of tendon degeneration and injury‐induced matrix turnover. Its sustained upregulation suggests that the tendon remains in a catabolic state, potentially degrading newly deposited matrix in an impaired attempt at remodeling [28]. *Mmp2* and *Mmp14*, also upregulated, support this conclusion, as they degrade basement membrane components and activate other MMPs, respectively [28, 29, 30]. The concurrent expression of multiple MMPs highlights a coordinated degradation program, likely initiated due to improperly formed or mechanically insufficient collagen networks [31]. *Loxl1* and *Loxl2*, enzymes responsible for catalyzing collagen crosslinking, were also upregulated. These enzymes typically act during ECM maturation to stiffen and stabilize fibrillar networks [32, 33, 34]. Their overexpression in TEN‐KO tendons likely reflects an attempt to reinforce structurally compromised fibrils, especially in the absence of proper diameter control during development. These changes are further supported by previous data demonstrating increased pyridinoline cross‐linking with knockout of collagen V in murine supraspinatus tendon [11, 35]. Several matricellular proteins were also upregulated in TEN‐KO tendons. *Postn* was highly upregulated in both the insertion and midsubstance. *Postn* promotes collagen fibrillogenesis and tenocyte mechanosensitivity and is known to be upregulated during tendon injury and healing [36, 37, 38]. Similarly, *Timp1*, an inhibitor of MMP activity, was also upregulated, likely representing a counter‐regulatory response to control matrix degradation [31]. Collectively, these changes highlight a mixed transcriptional response that includes both catabolic and anabolic signals—a hallmark of failed or maladaptive matrix repair. Notably, several genes were downregulated in TEN‐KO tendons. *Vegfb*, which promotes angiogenesis and metabolic support, was reduced, potentially limiting the tendon's regenerative capacity [39]. *Ltbp4*, which modulates TGF‐β bioavailability, was also downregulated, possibly dampening a key signaling axis for matrix synthesis [40].

In contrast, I‐NULL tendons displayed minimal transcriptional changes. The lack of broader gene expression changes supports the conclusion that collagen V is primarily required during tendon development, not maintenance. The mature tendon matrix appears to buffer against acute collagen V loss under homeostatic conditions, likely due to its low collagen turnover rate and stable fibrillar structure. These results also suggest that tenocytes in mature tendons are less transcriptionally responsive to changes in minor fibrillar collagens unless injury or inflammation is present. In total, these gene expression profiles support a model where developmental collagen V deficiency triggers a persistent state of maladaptive remodeling. Upregulation of MMPs, crosslinking enzymes, matricellular proteins, and signaling molecules reflect an unresolved injury‐like state, while loss of SLRPs and signaling factors limits the ability to restore matrix integrity. This transcriptomic phenotype aligns with mechanical, ultrastructural, and fiber realignment defects, suggesting that collagen V deficiency imprints a lasting molecular and functional deficit into the tendon.

These findings have important implications for our understanding of tendon development, homeostasis, and disease. In connective tissue disorders such as classic Ehlers‐Danlos syndrome (cEDS), where COL5A1 haploinsufficiency is common, the tendon‐related symptoms may stem primarily from developmental failures in matrix organization rather than ongoing matrix instability. This may explain the early onset and chronicity of joint hypermobility and tendon laxity in affected individuals [10] Furthermore, our results suggest that therapeutic efforts targeting collagen V expression may be most effective during development or early regenerative phases—times when matrix assembly is active and responsive.

Although our study provides important insights into the role of collagen V on supraspinatus tendon multiscale structure‐function relationships, it has certain limitations. One limitation is the absence of matrix compositional analysis. While we identified differential expression of key matrix genes, it remains unclear whether these transcriptional changes translate to alterations at the protein level. Future studies could employ proteomic approaches, such as nano‐flow liquid chromatography tandem mass spectrometry (LC‐MS/MS), to investigate differences in protein composition across genotypes [41]. Lastly, this study utilized male mice to reduce the impact of differences such as body weight and hormonal changes that may influence tendon health [42], particularly at this mature P150 timepoint. Future studies will expand on these findings to uncover potential sex‐dependent structural, functional, and compositional differences with collagen V deficiency and knockout.

In conclusion, our study reveals that collagen V plays a temporal role on supraspinatus tendon multiscale structure‐function. Its developmental expression is critical for organizing collagen fibrils, supporting proper mechanical adaptation, and establishing transcriptional homeostasis. Once assembled, the mature tendon ECM can persist without collagen V under homeostasis, as evident by minor changes in structure, function, and gene expression in the inducible tendons. While it is more than likely that orchestrated events between several minor collagens, proteoglycans, and glycoproteins are essential for both development and maintenance of tendon hierarchical structure [1], these findings have direct relevance to connective tissue disorders such as classic Ehlers‐Danlos Syndrome, where COL5A1 mutations produce lifelong joint laxity and tendon fragility. Therapeutic strategies aimed at collagen V should therefore be developmentally targeted or activated during regenerative windows when ECM remodeling is still active.

## Author Contributions

**Michael S. DiStefano** and **Jeremy D. Eekhoff:** data acquisition, analysis, interpretation, methodology, writing – original draft, writing – review and editing. **Stephanie N. Weiss:** mouse colony maintenance, sample collection, writing – review and editing. **Courtney A. Nuss** and **Rebecca L. Betts:** data acquisition, writing – review and editing. **Louis J. Soslowsky** and **Andrew F. Kuntz:** conceptualization, methodology, project administration, supervision, interpretation, writing – review and editing. All authors have read and approved the final submitted article.

## Supporting information

Table S1_Taqman assay information supplemental.

Table S2_Outliers.
